# Persistence of Neutralizing Antibodies to SARS-CoV-2 in First Wave Infected Individuals at Ten Months Post-Infection: The UnIRSA Cohort Study

**DOI:** 10.3390/v13112270

**Published:** 2021-11-12

**Authors:** Gloria Griffante, Shikha Chandel, Daniela Ferrante, Valeria Caneparo, Daniela Capello, Valentina Bettio, Cinzia Borgogna, Chiara Aleni, Salvatore Esposito, Andrea Sarro, Alessandra Vasile, Marco Comba, Tommaso Testa, Gianmarco Cotrupi, Marco De Andrea, Sara Bortoluzzi, Marisa Gariglio

**Affiliations:** 1 Department of Translational Medicine, University of Piemonte Orientale, 28100 Novara, Italy; gloria.griffante@uniupo.it (G.G.); shikha.chandel@uniupo.it (S.C.); daniela.ferrante@med.uniupo.it (D.F.); valeria.caneparo@med.uniupo.it (V.C.); daniela.capello@med.uniupo.it (D.C.); valentina.bettio@med.uniupo.it (V.B.); cinzia.borgogna@med.uniupo.it (C.B.); 20032401@studenti.uniupo.it (C.A.); 20017042@studenti.uniupo.it (S.E.); andrea.sarro@uniupo.it (A.S.); 20034878@studenti.uniupo.it (A.V.); marco.comba@uniupo.it (M.C.); tommaso.testa@uniupo.it (T.T.); 20034824@studenti.uniupo.it (G.C.);; 2 Center for Translational Research on Autoimmune and Allergic Disease (CAAD), University of Piemonte Orientale, 28100 Novara, Italy; marco.deandrea@unito.it; 3 UPO Biobank, University of Piemonte Orientale, 28100 Novara, Italy; 4 Department of Public Health and Pediatric Sciences, University of Turin, 10126 Turin, Italy

**Keywords:** SARS-CoV-2, COVID-19, neutralizing humoral response

## Abstract

Longitudinal mapping of antibody-based SARS-CoV-2 immunity is critical for public health control of the pandemic and vaccine development. We performed a longitudinal analysis of the antibody-based immune response in a cohort of 100 COVID-19 individuals who were infected during the first wave of infection in northern Italy. The SARS-CoV-2 humoral response was tested using the COVID-SeroIndex, Kantaro Quantitative SARS-CoV-2 IgG Antibody RUO Kit (R&D Systems, Bio-Techne, Minneapolis, USA) and pseudotype-based neutralizing antibody assay. Using sequential serum samples collected from 100 COVID-19 recovered individuals from northern Italy—mostly with mild disease—at 2 and 10 months after their first positive PCR test, we show that 93% of them seroconverted at 2 months, with a geometric mean (GeoMean) half-maximal neutralization titer (NT50) of 387.9. Among the 35 unvaccinated subjects retested at 10 months, 7 resulted seronegative, with an 80% drop in seropositivity, while 28 showed decreased anti-receptor binding domain (RBD) and anti-spike (S) IgG titers, with a GeoMean NT50 neutralization titer dropping to 163.5. As an NT50 > 100 is known to confer protection from SARS-CoV-2 re-infection, our data show that the neutralizing activity elicited by the natural infection has lasted for at least 10 months in a large fraction of subjects.

## 1. Introduction

Severe acute respiratory syndrome coronavirus 2 (SARS-CoV-2) infection is associated with the development of variable levels of antibodies (Abs) with neutralizing activity, which can protect against infection in animal models [1,2,3]. However, the duration of the serological response in infected subjects and the extent to which such Ab response may be protective against reinfection [4] have still to be fully characterized.

Given the short time SARS-CoV-2 has been studied, information on long-term antibody dynamics is still limited. In this regard, quantitative titer determination of virus-neutralizing Abs (nAbs) is considered an excellent correlate of protection (CoP). Initial reports pointed to a rapid decline in the humoral response within 3–4 months post-infection [5,6,7]. More recently, longitudinal studies assessing mid-term kinetics of SARS-CoV-2 infection showed persistent neutralizing antibody responses for up to 8–10 months [8,9,10], leading to the possibility that nAbs to SARS-CoV-2 may also represent a CoP from emerging SARS-CoV-2 variants of concern.

The aim of this study was to assess the longitudinal profile of anti-spike (S) IgG and anti-recombinant receptor binding domain (RBD) Abs as well as the SARS-CoV-2 neutralizing activity of sera from 100 individuals who were infected during the first wave of SARS-CoV-2 infection in Italy. Although a decline in both IgG levels and neutralizing activity was observed overtime, most of the study subjects still retained a neutralizing activity above the cut-off value (i.e., GeoMean NT50, the reciprocal dilution inhibiting 50% of the infection) of 100, which is considered to be effective in reducing the risk of reinfection [11,12].

## 2. Material and Methods

### 2.1. Ethical Statement

Participants were involved and consented under the UPO Biobank study and ethical governance approved by the Ethics Committee of “Maggiore della Carità” Hospital (protocol 427/CE; study No. CE 84/20).

### 2.2. Human Subjects, Sample, and Data Collection

Blood samples were obtained from individuals enrolled in the UnIRSA (Unveiling the Immune Response against SARS-CoV-2) cohort study. An observational study was carried out on a cohort of 100 individuals with real-time reverse-transcriptase-polymerase chain reaction (rRT-PCR)-confirmed SARS-CoV-2 infection from nasopharyngeal swab that dated from 14 March 2020 to 17 May 2020, thus corresponding to the first wave of infection in Italy. Patient recruitment commenced in May 2020 and continued until June 2020. Recruited patients were followed up until February 2021. The inclusion criteria were (i) one positive rRT-PCR test for SARS-CoV-2 followed by two consecutive negative tests performed 24 h apart, that fulfilled the administrative definition of recovery from COVID-19 as established by the Italian Ministry of Health on 28 February 2020, (ii) age ≥ 18 years, (iii) absence of an immunosuppressive conditions, and (iv) willingness to provide an informed consent. At blood sample collection, an ad-hoc questionnaire was administered to collect data, including socio-demographic characteristics, biometric data (e.g., weight, height, body mass index), and presentation of SARS-CoV-2 infection.

The study participants were stratified according to COVID-19 disease severity as follows: (i) asymptomatic patients, reporting no symptoms, (ii) symptomatic patients, disclosing at least one symptom of those indicated in Table 1 without the need of hospitalization, and (iii) hospitalized patients, requiring hospital assistance due to the severity of COVID-19.

A retrospective chart review was performed on hospitalized participants. Samples were coded and then de-identified prior to analysis. Other efforts to maintain the confidentiality of participants consisted in labeling samples with coded identification numbers. All the data were recorded on the REDCap (https://www.project-redcap.org/, accessed on 8 November 2021) web application in compliance with current General Data Protection Regulation (GDPR) and Italian legislation on the protection of sensitive data and privacy.

### 2.3. Quantitative Determination of Anti-SARS-CoV-2-Specific Abs

To perform quantitative determination of anti-SARS-CoV-2 Abs, the COVID-SeroIndex, Kantaro Quantitative SARS-CoV-2 IgG Antibody RUO Kit (R&D Systems, Bio-Techne, Minneapolis, MN, USA), which comprises two serial direct enzyme-linked immunosorbent assays (ELISA), was employed. The immunoassays were used and interpreted following the manufacturer’s instructions. Briefly, an initial ELISA was performed to test the reactivity of Abs raised against the recombinant receptor binding domain (RBD) of the SARS-CoV-2 spike protein from the first virus isolate Wuhan-Hu-1 [13,14]. Results were expressed as cut-off index (CI) calculated as the ratio of the corrected sample OD value to the corrected positive control optical density (OD) value. CI values > 0.7 were considered positive. In the quantitative ELISA against the full-length SARS-CoV-2 spike protein (S), the Ab concentration of samples was calculated using a four-parameter logistic (4-PL) curve fit. Values below the limit of quantification (LoQ) of 3.2 AU/mL were considered negative.

### 2.4. SARS-CoV-2-Specific Neutralizing Antibody Assay

Vero E6 and Vero E6-TMPRSS2—kindly provided by John Hiscott, Pasteur Institute Rome—were cultured in Dulbecco’s modified Eagle’s medium (DMEM) supplemented with 10% fetal calf serum (FCS) (Sigma-Aldrich, Milan, Italy). The replication-competent vesicular stomatitis virus r(VSV)-eGFP-SARS-CoV-2-SΔ21 was a kind gift from Sean P.J. Whelan (Washington University School of Medicine, USA) [15]. To grow this virus, Vero E6 cells were infected with a low Multiplicity of Infection (MOI) (0.01) and maintained at 34 °C from then on. Cell supernatants were harvested upon visualization of extensive cytopathic effect and cell detachment at approximately 24 h post-infection (hpi). Upon viral RNA extraction and amplification by RT-PCR, the *spike* gene was sequenced every time the virus was harvested. The virus was titrated by flow cytometry. Serum samples were heat-inactivated at 56 °C for 30 min. Indicated dilutions of sera were incubated with rVSV-SARS-CoV-2-SΔ21 at an MOI of 0.05 for 1 h at 37 °C. Ab-virus complexes were added to Vero E6-TMPRSS2 cells in 96-well plates and incubated at 34 °C for 24 h. Subsequently, cells were fixed in 4% formaldehyde (Millipore Sigma, Burlington, MA, USA) containing DAPI for 15 min on ice, when fixative was replaced with PBS. Images were acquired using Cytation 5 Cell Imaging Multi-Mode Reader (BioTek, Winooski, VT, USA) in both the DAPI and GFP channels to visualize nuclei and infected cells (i.e., eGFP-positive cells). The raw images (2 × 2 montage) were acquired using 4X objective, processed, and stitched using the default setting. 

### 2.5. Statistical Analysis

Normally distributed data were represented as mean and standard deviation (SD), whereas data following a non-normal distribution were represented as median and interquartile range (IQR). Categorical variables were summarized as counts and percentages. Differences in medians were evaluated using the Mann–Whitney’s U test and Wilcoxon Rank signed-rank test for pairwise comparisons. The Kruskal–Wallis test along with the Dunn test for multiple comparisons was used to compare more than two groups. The Bonferroni correction method was applied.

The Spearman’s correlation coefficient was used to compute correlation between quantitative variables. A two-sided *p*-value < 0.05 was considered statistically significant. Images and raw data were processed, and the NT50 (the reciprocal dilution inhibiting 50% of the infection) was calculated by plotting and fitting the log of serum dilution versus response to a 4-parameters equation using the Gen5 v3.0 software. Data were processed using Prism software (GraphPad Prism v6.0) and STATA v16 (College Station, TX, USA).

## 3. Results

### 3.1. Patient Selection and Magnitude of the Antibody Response to SARS-CoV-2 at Two Months after the First Positive PCR Test (M2)

From 21 May to 19 June 2020, 100 individuals (77 female, 23 male) who were infected by SARS-CoV-2 during the first wave of infection in Italy were enrolled in this study (Table 1). Their median age was 46.5 years (IQR 33.5,52.8). All study subjects had an RT-PCR-confirmed SARS-CoV-2 infection. While most of these COVID-19 patients (74%) experienced mild-to-moderate disease or did not report any symptoms (16%), 10% of them required hospitalization without the need of intensive care unit (ICU)-level care.

**Figure 1:**
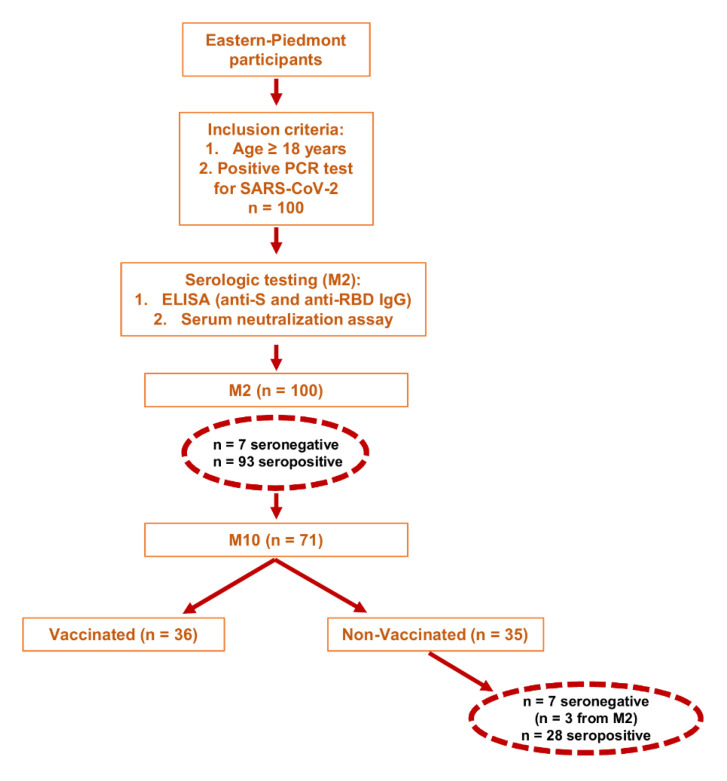
Study design and participants.

All patients provided an initial blood sample defined as M2 (approximately two months post-infection), which was taken at a median number of 51 days (interquartile range, IQR 43, 56) from the first positive PCR test. A second longitudinal blood sample, defined as M10 (approximately 10 months post-infection), was taken from 71 COVID-19 patients at a median number of 293 days after the initial sample collection (IQR 287,303 days). During the observation period, the Italian COVID-19 vaccine campaign started and, between 2 January 2021 and 4 February 2021, 21 study subjects received a single dose of mRNA-based SARS-CoV-2 vaccines, whereas 15 obtained two doses, bringing the total number of study subjects with at least one dose to 36 (Figure 1).

**Figure 2:**
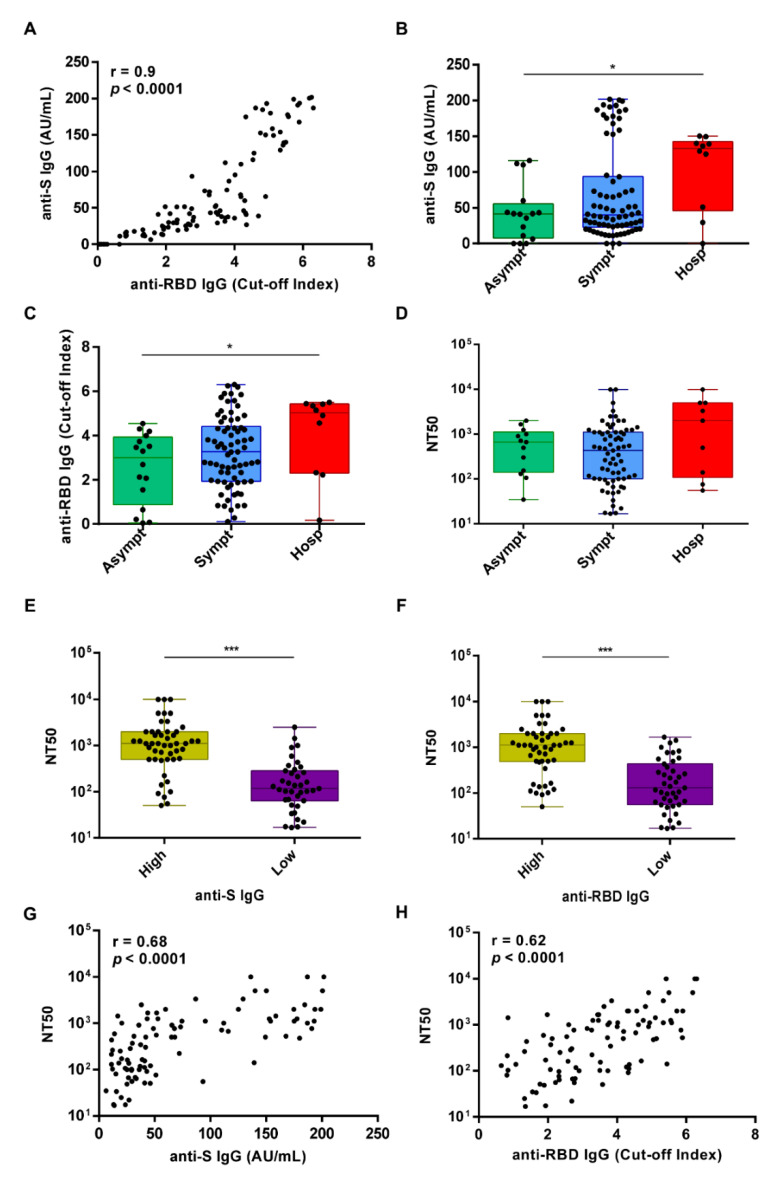
Antibody responses in the study cohort following SARS-CoV-2 infection at 1.5 months (M2) after the first positive PCR test. (**A**) Spearman’s correlation between anti-S IgG AU/mL and anti-RBD IgG cut-off index as assessed by ELISA at M2. (**B**–**D**) The 100 study subjects were categorized into 3 groups according to disease severity (asymptomatic n = 16, symptomatic n = 74, or hospitalized n = 10) and plotted according to anti-S IgG AU/mL (**B**) or anti-RBD IgG cut-off index (**C**) and the neutralization titer expressed as NT50 (**D**). Solid circles indicate individual values. The *p*-value among different groups was calculated by Kruskal–Wallis test * *p* < 0.05. (**E**) Box plot distribution of the neutralizing activity expressed as NT50 in the group of high or low anti-S IgG levels—above and below the median, respectively. (**F**) Box plot distribution of the neutralizing activity expressed as NT50 in the group of high or low anti-RBD IgG levels—above and below the median, respectively. In E and F, the solid circles indicate individual values. *p*-values were determined by two-sided Mann-Whitney test. *** *p* < 0.001. (**G**) Spearman correlation between the neutralization titer expressed as NT50 and the anti-S IgG levels. (**H**) Spearman correlation between the neutralization titer expressed as NT50 and the anti-RBD IgG levels.

Circulating IgG Abs against the SARS-CoV-2 spike antigen were measured using the Kantaro Quantitative SARS-CoV-2 IgG Antibody RUO Kit, which allows detecting IgG against either the RBD or the full-length trimeric spike protein of SARS-CoV-2. At M2, 93% of the subjects showed detectable levels of IgG Abs against both RBD and total S protein, with a positive spearman correlation (r = 0.9, *p* < 0.0001) (Figure 2A). In addition, we identified 7 subjects, one of whom had been hospitalized for pneumonia, lacking circulating Abs to SARS-CoV-2 (Table 2). Three of them were retested at M10 but remained seronegative (Figure 1).

After patient stratification for COVID-19 disease severity (i.e., asymptomatic, symptomatic, or hospitalized), we observed that both anti-S and anti-RBD Ab levels were significantly higher in the group of hospitalized patients than those detected in asymptomatic cases (*p* = 0.04 and *p* = 0.03, respectively) (Figure 2B,C). A similar trend was observed for the neutralizing titers although not statistically significant (*p =* 0.12) (Figure 2D).

Given that the determination of the neutralizing effects of SARS-CoV-2 spike Abs is critical to understand the protective effects of the immune response, the same sera were also analyzed for their ability to inhibit VSV-SARS-CoV-2-SΔ21 infection of Vero E6 cells, as previously described [15,16]. The latter is a very useful BSL2 surrogate virus whose neutralization profiles strongly correlate with focus-reduction neutralization tests using SARS-CoV-2. We observed that sera with values above the median titer for both anti-S and anti-RBD IgG displayed significantly higher neutralizing activity when compared to those with values below the median value (*p* < 0.0001 for both) (Figure 2E,F), with a positive Spearman correlation r = 0.68, *p* < 0.0001 and r = 0.62, *p* < 0.0001, respectively (Figure 2G,H). The geometric mean (GeoMean) NT50 neutralization titer was 387.9 (CI 95% 280.8, 535.8) at M2, with 7% of individuals not reaching 50% neutralization at the lowest serum dilution of 1:10.

### 3.2. Comparative Analysis of the IgG Titers and the Neutralizing Response at Ten Months after the First Positive PCR Test (M10)

Seventy-one subjects provided additional blood samples at M10, which were assessed for the presence of both anti-S and anti-RBD IgG as described for the M2 samples. Notably, at M10, 36 study subjects had received at least one dose of mRNA-based SARS-CoV-2 vaccines.

**Figure 3:**
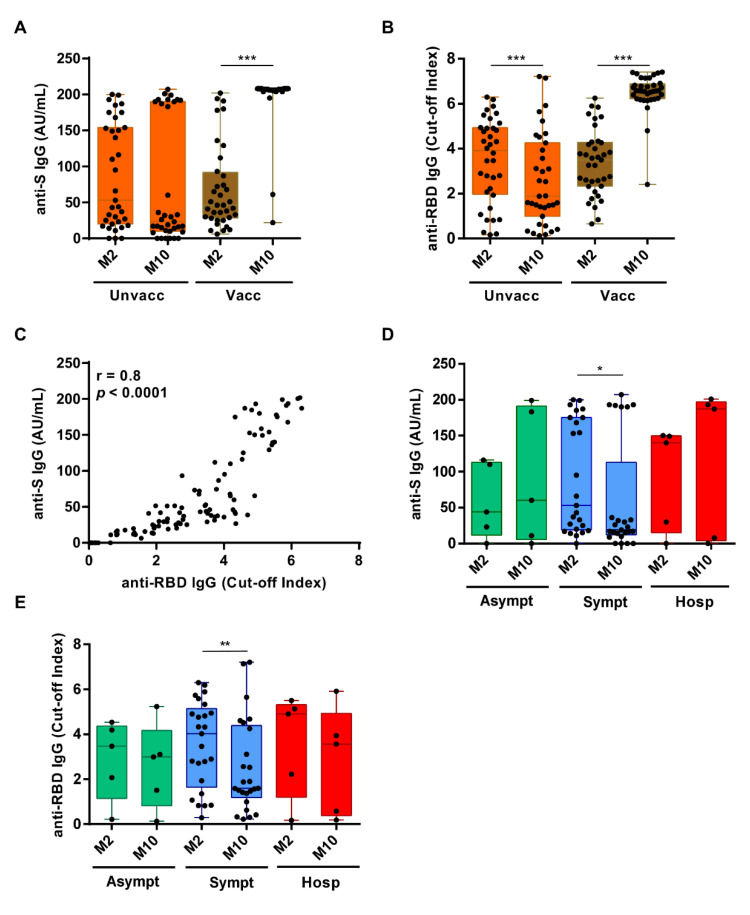
Dynamic changes in anti-S and anti-RBD IgG levels between M2 and M10 after the first PCR-positive test. (**A**) Box plot distribution of anti-S IgG levels or (**B**) anti-RBD IgG levels in unvaccinated (left) or vaccinated (right) subjects at M2 vs. M10. Solid circles indicate individual values. *p*-values were calculated by two-sided Wilcoxon signed-rank tests. *** *p* < 0.001. (**C**) Spearman correlation between anti-S IgG AU/mL and anti-RBD IgG cut-off index as assessed by ELISA at M10. (**D**) Comparison of anti-S IgG levels between asymptomatic (n = 16), symptomatic (n = 74), or hospitalized (n = 10) subjects at M2 and M10. *p*-values were calculated by two-sided Wilcoxon signed-rank tests. * *p* < 0.05. (**E**) Comparison of anti-RBD cut-off index between asymptomatic n = 16, symptomatic n = 74, or hospitalized n = 10 subjects at M2 or M10. *p*-values were calculated by two-sided Wilcoxon signed-rank test. ** *p* < 0.01.

As the objective of this study was to determine the duration of the immune response to natural infection, assessment of nAbs was only performed for those unvaccinated subjects that had positive ELISA IgG Ab test results (n = 28/35). As expected, the IgG titers against S and RBD antigens were both significantly increased in vaccinated subjects at M10 vs. M2 (*p* < 0.0001 for both Abs) (Figure 3A,B, respectively). By contrast, in the 35 unvaccinated individuals the scenario was completely different: 28 of them remained seropositive for both anti-S and anti-RBD Ab, with a positive Spearman correlation r = 0.8, *p* < 0.0001 (Figure 3C), while 7 turned out to be seronegative. Three of these latter were already seronegative at M2, whereas the other 4 lost their anti-S and anti-RBD IgG reactivity overtime. Specifically, the percentage of subjects seropositive for both anti-S and anti-RBD IgG within this unvaccinated cohort was 91% (32/35) at M2 vs. 80% (28/35) at M10. When we assessed the levels of the Abs over time, we found that the anti-RBD Abs had significantly decreased between M2 and M10 (*p* = 0.0007), while the anti-S Abs had not significantly changed (*p* = 0.38) (Figure 3A,B, respectively). However, when the subjects were stratified for COVID-19 symptoms, also the decrease in anti-S Abs in symptomatic patients became statistically significant (*p* = 0.03), while it remained unchanged in the other two subgroups (i.e., hospitalized and asymptomatic). A similar trend was also observed for the anti-RBD Ab titers (*p* = 0.003) (Figure 3D,E, respectively).

**Figure 4:**
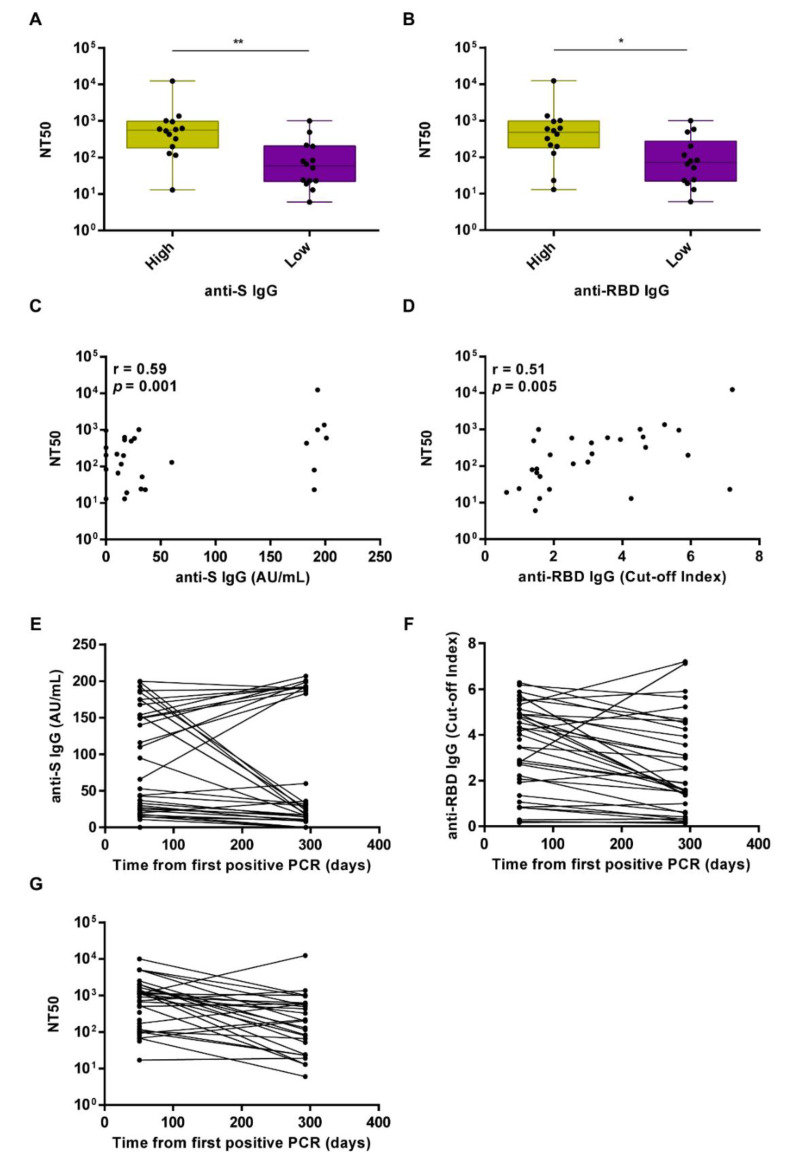
Longitudinal analysis of the neutralizing activity at 10 months (M10) after the first positive PCR test. (**A**) Box plot distribution of the neutralizing activity expressed as NT50 in the group of high or low anti-S IgG levels—above and below the median, respectively. Comparative analysis was performed by Mann-Whitney test. ** *p* < 0.01. (**B**) Box plot distribution of the neutralizing activity expressed as NT50 in the group of high or low anti-RBD IgG levels—above and below the median, respectively. Comparative analysis was performed by Mann-Whitney test. * *p* < 0.05. Solid circles indicate individual values. (**C**) Spearman correlation between anti-S IgG AU/mL and the neutralizing activity expressed as NT50 at M10. (**D**) Spearman correlation between anti-RBD IgG cut-off index and the neutralizing activity expressed as NT50 at M10. (**E**) Longitudinal mapping of anti-RBD IgG cut-off index, anti-S IgG AU/mL (**F**), or neutralizing activity expressed as NT50 (**G**) in seropositive unvaccinated subjects (n = 28) at M2 and M10 after the first PCR-positive test.

With regard to the neutralizing Ab titers at M10, we observed that sera displaying values above the median titer for the anti-S IgG displayed significantly higher neutralizing activity compared to that of sera with anti-S values below the median value (*p* = 0.0028) (Figure 4A). When we considered anti-RBD IgG titers below and above the median value, the trend was similar (*p* = 0.0131) (Figure 4B). The positive Spearman correlation between anti-S or anti-RBD titers and nAb titers was r = 0.59 (*p* = 0.001) and r = 0.51 (*p* = 0.005), respectively, indicating an overall lower correlation when compared to that observed at M2 (Figure 4C,D, respectively). The GeoMean NT50 neutralization titer was 163.5 (CI 95% 82.1, 325.9) at M10.

We next measured and correlated anti-S, anti-RBD IgG, and nAb levels in the sera of the 28 individuals who had not received the vaccine and remained seropositive at M10. While most of them displayed reduced anti-RBD IgG (n = 22) and nAb levels (n = 21) at M10 vs. M2, 8 unvaccinated patients still retained nAb titers above the 1:100 cut-off at M10 (48%). On the other hand, 6 subjects showed increased anti-RBD IgG levels at M10 vs. M2, and 6 displayed increased nAb titer values above the 1:100 cut-off at M10 vs. M2, while 1 subject retained low but sustained nAb titer values below the 1:100 cut-off. When we compared anti-S IgG levels at M10 with those at M2, we found them to be reduced in 16 subjects and upregulated in 12 (Figure 4E–G). Interestingly, we found that those who had increased anti-S IgG levels at M10 were older when compared to those who displayed decreased anti-S IgG levels at M10 (*p* = 0.01), as already reported in a series of studies conducted in healthy subjects [17,18,19]. Next, using the mathematical modeling approach developed by Miles P. Davenport and co-workers, which provides a quantitative prediction of the link between neutralizing antibody levels and clinical protection, we estimated the 50% protective neutralization level against SARS-CoV-2 infection in our cohort to be 78.338 in the M2 group, calculated as 20.2% of the mean level [20]. Using this predictive model and threshold, we found that 80% (28/35) of the subjects in the M2 group and 54.28% (19/35) in the M10 group were above this value. Overall, our findings show that 60% of the subjects in the unvaccinated cohort (n = 35) experienced a decline in their serum neutralizing activity at M10, while 20% did show increased nAb levels over time. The antibody levels were below the limit of detection in the remaining 20% of the subjects, indicating that 11.5% completely lost the humoral response, while 8.5% never mounted an immune response.

## 4. Discussion

In this study, we have performed a longitudinal analysis of the serological responses to SARS-CoV-2 in 100 COVID-19 patients who were infected during the first wave of infection in Italy, of whom 74 were HCWs working at a hospital setting in northern Italy. In these patients, we performed quantitative determination of the anti-RBD and anti-S IgG response to SARS-CoV-2 and evaluated the neutralizing activity of their sera using an in vitro functional assay.

Both anti-RBD and anti-S antibody levels were below the detection limit of the assays in 7% of the subjects after ~50 days from the first SARS-CoV-2 positive PCR test, indicating that these patients did not seroconvert or had already lost their seroconversion at this time point [5,19,21]. At this time point, both anti-RBD and anti-S IgG levels were significantly higher in hospitalized subjects than those observed in asymptomatic or symptomatic patients. Among the 35 subjects who did not receive the vaccine between the M2 and M10 time points, 4 (11%) became seronegative with a drop in seropositivity from 91% at 2 to 80% at 10 months after SARS-CoV-2 infection. Most of the patients displayed lower IgG levels against both S and RBD at M10 vs. M2 (16/35 (42.7%) and 22/35 (62.8%), respectively). Accordingly, the GeoMean NT50 neutralizing titer dropped from 387.9 at M2 to 163.5 at M10. Likewise, the positive Spearman correlation between anti-S or anti-RBD IgG levels and the neutralizing activity of the sera decreased during the interval from M2 to M10, suggesting that the decline in anti-S and RBD IgG levels at longer time points post-infection may not faithfully reflect a similar decline in their neutralizing activity. Recent longitudinal studies aimed at investigating the duration of humoral immune response in COVID-19-recovered individuals reported similar decay kinetics. Overall, the decline appears to occur up to 7–9 months while it is thereafter stabilized at least until 12 months [19,22,23,24].

SARS-CoV-2 neutralizing Abs are excellent CoP because they can exert their antiviral activity by acting at the mucosal surface, which curbs the initial infection, mostly mediated by secretory IgA whose levels were shown to rise early after natural infection and neutralize the virus even to a greater extent than IgG [25]. In addition, in the bloodstream as circulating IgM or IgG, they block subsequent viral spread and protect from disease progression. Furthermore, by slowing down the growth rate of SARS-CoV-2, these nAbs may also favor the recruitment of memory B cells capable of neutralizing the infection [26]. Fittingly, vaccine-induced nAbs as well as purified IgG from convalescent animals have been shown to protect non-human primates (NHPs) from infection in a SARS-CoV-2 challenge model [1,2].

One of the major issues in assessing whether nAbs are good proxies for protection from SARS-CoV-2 infection is the determination of a titer cut-off value or range that would allow the identification of those subjects with enough neutralizing activity to make them resistant or less susceptible to reinfection. In spike-based mRNA vaccines studies among people aged 18–55, the GeoMean neutralizing titers after the second dose was 1:181 (day 85), or 1:430 (day 119) depending on the neutralization assay used, while an adenovirus-based vaccine gave an NT50 value of 1:161 or 1:193 (day 42) depending on the neutralization assay used [12]. Another study involving subjects from high attack rate events reported that neutralizing activities in the range 1:100–1:200 were strong enough to prevent infection [11]. Although direct comparisons among the aforementioned studies may suffer from some bias due to the use of different neutralization assays [8,9,15], the emerging concept from this recent body of literature is that an NT50 neutralization titer > 100 is likely to confer protection from SARS-CoV-2 re-infection [11], [reviewed in 12]. Thus, in this context, the fact that we detected a GeoMean NT50 titer of 163.5 at 10 months after the first SARS-CoV-2 positive PCR test would indicate that a significant proportion of individuals (48%) were still within the GeoMean range observed in the vaccinated cohorts. This would suggest that the neutralizing activity elicited by the natural infection may still be effective in a large fraction of subjects (17/35 in our study) after 10 months from the initial infection, even after experiencing a very mild form of COVID-19 disease. More specifically, we found that 74/100 (74%) subjects displayed NT50 neutralizing titer >100 at M2, while in the non-vaccinated subgroup it was 26/35 at M2 (74%) and 17/35 at M10 (48%). In addition, according to the mathematical model developed by Khoury et al., 80% of the subjects at M2 and 54.2% at M10 displayed a neutralization titer that was deemed to be sufficient to provide 50% protection from symptomatic COVID-19 [20].

Our analysis is limited by the reduced sample size, particularly at M10, as half of the study participants received the vaccine in the frame of the Italian vaccination campaign which started during the observational period. However, despite the limited sample size, the availability of two longitudinal measurements, one of which up to 10 months after initial diagnosis, allowed us to achieve adequate statistical power to establish that the neutralizing antibody titer against SARS-CoV-2 were still present in 80% of the study subjects who were infected during the first wave of SARS-CoV-2 infection in Italy. Another limitation of this study is that it is possible that the trends of immune response in recovered, non-vaccinated patients might reflect re-exposure to SARS-CoV-2, particularly in those for whom titers augmented with time. We need also to point out that the requirement for fulfilling the administrative definition of recovery by COVID-19 (as previously described in Material and Methods) provided a homogenizing filter, and thus patients who had lingering detectable SARS-CoV-2 may behave differently. Another limitation is that the role of the memory B cell compartment has not been analyzed in this study. Indeed, the number of RBD-specific memory B cells has been reported to remain stable between 6 and 12 months upon natural infection [19].

Finally, the recombinant VSV expressing SARS-CoV-2 S protein was shown to behave analogously to a clinical isolate of SARS-CoV-2 and to provide comparable results to neutralization tests with the wild-type virus [15]. The r(VSV)-eGFP-based SARS-CoV-2 neutralizing Ab assay may offer utility as a diagnostic tool with which to assess patients’ sera neutralizing activity. We postulate it could be predictive of the likelihood of reinfection in the general population.

## Figures and Tables

**Table 1:** Characteristics of the study population with SARS-CoV-2 infection.

	n = 100
Age at COVID-19 diagnosis (years) median (IQR)	46.5 (33.5, 52.8)
BMI, kg/m^2^ Median (IQR)	24.2 (21.9, 28.1)
Sex, n (%)	
female	77 (77)
male	23 (23)
Occupation, n (%)	
Physician or paramedical staff	74 (74)
Others	26 (26)
Comorbidities, n (%)	
No comorbidities	53 (53)
allergy	29 (29)
hypertension	14 (14)
autoimmune diseases	13 (3)
asthma	5 (5)
cancer	3 (3)
diabetes	3 (3)
heart disease	2 (2)
Reported Symptoms, n (%)	
No symptoms	16 (16)
asthenia	57 (57)
anosmia	55 (55)
muscle ache	53 (53)
fever	52 (52)
ageusia	49 (49)
headache	47 (47)
cough	44 (44)
diarrhea	35 (35)
runny nose	28 (28)
dyspnea	24 (24)
chest pain	22 (22)
skin manifestations	14 (14)
palpitations	13 (13)
Severity, n (%)	
asymptomatic	16 (16)
symptomatic	74 (74)
hospitalized	10 (10)
Median (IQR) days between positive PCR and first antibody test (M2)	51 (43, 56)
Median (IQR) days between positive PCR and second antibody test (M10)	293 (287, 303)
Vaccinated (among 71 subjects with second antibody test), n (%)	36 (50.7)
One dose, n (%)	21 (29.6)
Two doses, n (%)	15 (21.1)
Median (IQR) days between vaccination (single dose) and second antibody test (M10)	15 (11, 18)
Median (IQR) days between vaccination (second dose) and second antibody test (M10)	8 (3, 10)

**Table 2:** Clinical and laboratory characteristics of the seronegative individuals at M2.

Subject	Age	Gender	Symptoms	Comorbidities	M2	M10
1	30	F	Asymptomatic	None	55	NA
2	36	M	Symptomatic	None	50	NA
3	63	F	Symptomatic	Cancer	43	NA
4	27	F	Asymptomatic	Allergy	43	NA
5	52	F	Pneumonia	None	43	232
6	58	F	Asymptomatic	None	48	246
7	31	F	Symptomatic	None	27	254

NA, not available.

## Data Availability

The data presented in this study are available on request from the corresponding author.

## References

[1] Mercado N.B., Zahn R., Wegmann F., Loos C., Chandrashekar A., Yu J., Liu J., Peter L., Mcmahan K., Tostanoski L.H. (2020). Single-shot Ad26 vaccine protects against SARS-CoV-2 in rhesus macaques. Nature.

[2] McMahan K., Yu J., Mercado N.B., Loos C., Tostanoski L.H., Chandrashekar A., Liu J., Peter L., Atyeo C., Zhu A. (2021). Correlates of protection against SARS-CoV-2 in rhesus macaques. Nat. Cell Biol..

[3] Robbiani D.F., Gaebler C., Muecksch F., Lorenzi J.C.C., Wang Z., Cho A., Agudelo M., Barnes C.O., Gazumyan A., Finkin S. (2020). Convergent antibody responses to SARS-CoV-2 in convalescent individuals. Nat. Cell Biol..

[4] Röltgen K., Powell A.E., Wirz O.F., Stevens B.A., Hogan C.A., Najeeb J., Hunter M., Wang H., Sahoo M.K., Huang C. (2020). Defining the features and duration of antibody responses to SARS-CoV-2 infection associated with disease severity and outcome. Sci. Immunol..

[5] Marot S., Malet I., Leducq V., Zafilaza K., Sterlin D., Planas D., Gothland A., Jary A., Dorgham K., Bruel T. (2021). Rapid decline of neutralizing antibodies against SARS-CoV-2 among infected healthcare workers. Nat. Commun..

[6] Yamayoshi S., Yasuhara A., Ito M., Akasaka O., Nakamura M., Nakachi I., Koga M., Mitamura K., Yagi K., Maeda K. (2021). Antibody titers against SARS-CoV-2 decline, but do not disappear for several months. EClinicalMedicine.

[7] Gaebler C., Wang Z., Lorenzi J.C.C., Muecksch F., Finkin S., Tokuyama M., Cho A., Jankovic M., Schaefer-Babajew D., Oliveira T.Y. (2021). Evolution of antibody immunity to SARS-CoV-2. Nature.

[8] Dan J.M., Mateus J., Kato Y., Hastie K.M., Yu E.D., Faliti C.E., Grifoni A., Ramirez S.I., Haupt S., Frazier A. (2021). Immunological memory to SARS-CoV-2 assessed for up to 8 months after infection. Science.

[9] Pradenas E., Trinité B., Urrea V., Marfil S., Ávila-Nieto C., de la Concepción M.L.R., Tarrés-Freixas F., Pérez-Yanes S., Rovirosa C., Ainsua-Enrich E. (2021). Stable neutralizing antibody levels 6 months after mild and severe COVID-19 episodes. Med.

[10] Levi R., Ubaldi L., Pozzi C., Angelotti G., Sandri M.T., Azzolini E., Salvatici M., Savevski V., Mantovani A., Rescigno M. (2021). The antibody response to SARS-CoV-2 infection persists over at least 8 months in symptomatic patients. Commun. Med..

[11] Addetia A., Crawford K.H.D., Dingens A., Zhu H., Roychoudhury P., Huang M.-L., Jerome K.R., Bloom J.D., Greninger A.L. (2020). Neutralizing antibodies correlate with protection from SARS-CoV-2 in humans during a fishery vessel outbreak with a high attack rate. J. Clin. Microbiol..

[12] Koch T., Mellinghoff S., Shamsrizi P., Addo M., Dahlke C. (2021). Correlates of Vaccine-Induced protection against SARS-CoV-2. Vaccines.

[13] Wu F., Zhao S., Yu B., Chen Y.-M., Wang W., Song Z.-G., Hu Y., Tao Z.-W., Tian J.-H., Pei Y.-Y. (2020). A new coronavirus associated with human respiratory disease in China. Nature.

[14] Amanat F., Stadlbauer D., Strohmeier S., Nguyen T.H.O., Chromikova V., McMahon M., Jiang K., Arunkumar G.A., Jurczyszak D., Polanco J. (2020). A serological assay to detect SARS-CoV-2 seroconversion in humans. Nat. Med..

[15] Case J.B., Rothlauf P.W., Chen R.E., Liu Z., Zhao H., Kim A.S., Bloyet L.-M., Zeng Q., Tahan S., Droit L. (2020). Neutralizing Antibody and Soluble ACE2 Inhibition of a Replication-Competent VSV-SARS-CoV-2 and a Clinical Isolate of SARS-CoV-2. Cell Host Microbe.

[16] Borgogna C., De Andrea M., Griffante G., Lai A., Bergna A., Galli M., Zehender G., Castello L., Ravanini P., Cattrini C. (2021). SARS-CoV-2 reinfection in a cancer patient with a defective neutralizing humoral response. J. Med. Virol..

[17] Vanshylla K., Di Cristanziano V., Kleipass F., Dewald F., Schommers P., Gieselmann L., Gruell H., Schlotz M., Ercanoglu M.S., Stumpf R. (2021). Kinetics and correlates of the neutralizing antibody response to SARS-CoV-2 infection in humans. Cell Host Microbe.

[18] Glück V., Grobecker S., Tydykov L., Salzberger B., Glück T., Weidlich T., Bertok M., Gottwald C., Wenzel J.J., Gessner A. (2021). SARS-CoV-2-directed antibodies persist for more than six months in a cohort with mild to moderate COVID-19. Infection.

[19] Li C., Yu D., Wu X., Liang H., Zhou Z., Xie Y., Li T., Wu J., Lu F., Feng L. (2021). Twelve-month specific IgG response to SARS-CoV-2 receptor-binding domain among COVID-19 convalescent plasma donors in Wuhan. Nat. Commun..

[20] Khoury D.S., Cromer D., Reynaldi A., Schlub T.E., Wheatley A.K., Juno J.A., Subbarao K., Kent S.J., Triccas J.A., Davenport M.P. (2021). Neutralizing antibody levels are highly predictive of immune protection from symptomatic SARS-CoV-2 infection. Nat. Med..

[21] Wajnberg A., Amanat F., Firpo A., Altman D.R., Bailey M.J., Mansour M., McMahon M., Meade P., Mendu D.R., Muellers K. (2020). Robust neutralizing antibodies to SARS-CoV-2 infection persist for months. Science.

[22] Vacharathit V., Srichatrapimuk S., Manopwisedjaroen S., Kirdlarp S., Srisaowakarn C., Setthaudom C., Inrueangsri N., Pisitkun P., Kunakorn M., Hongeng S. (2021). SARS-CoV-2 neutralizing antibodies decline after one year and patients with severe COVID-19 pneumonia display a unique cytokine profile. Int. J. Infect. Dis..

[23] Petersen M.S., Hansen C.B., Kristiansen M.F., Fjallsbak J.P., Larsen S., Hansen J.L., Jarlhelt I., Pérez-Alós L., Steig B.Á., Christiansen D.H. (2021). SARS-CoV-2 natural antibody response persists for at least 12 months in a nationwide study from the Faroe Islands. Open Forum Infect. Dis..

[24] Wang Z., Muecksch F., Schaefer-Babajew D., Finkin S., Viant C., Gaebler C., Hoffmann H.-H., Barnes C.O., Cipolla M., Ramos V. (2021). Naturally enhanced neutralizing breadth against SARS-CoV-2 one year after infection. Nat. Cell Biol..

[25] Sterlin D., Mathian A., Miyara M., Mohr A., Anna F., Claër L., Quentric P., Fadlallah J., Devilliers H., Ghillani P. (2021). IgA dominates the early neutralizing antibody response to SARS-CoV-2. Sci. Transl. Med..

[26] Corti D., Lanzavecchia A. (2013). Broadly Neutralizing Antiviral Antibodies. Annu. Rev. Immunol..

